# Atom-based machine learning for estimating nucleophilicity and electrophilicity with applications to retrosynthesis and chemical stability†

**DOI:** 10.1039/d4sc07297a

**Published:** 2025-02-25

**Authors:** Nicolai Ree, Jan M. Wollschläger, Andreas H. Göller, Jan H. Jensen

**Affiliations:** a Department of Chemistry, University of Copenhagen Universitetsparken 5 2100 Copenhagen Ø Denmark jhjensen@chem.ku.dk; b Bayer AG, Pharmaceuticals, R&D, Machine Learning Research 13353 Berlin Germany; c Bayer AG, Pharmaceuticals, R&D, Computational Molecular Design 42096 Wuppertal Germany andreas.goeller@bayer.com

## Abstract

Nucleophilicity and electrophilicity are important properties for evaluating the reactivity and selectivity of chemical reactions. It allows the ranking of nucleophiles and electrophiles on reactivity scales, enabling a better understanding and prediction of reaction outcomes. Building upon our recent work (N. Ree, A. H. Göller and J. H. Jensen, Automated quantum chemistry for estimating nucleophilicity and electrophilicity with applications to retrosynthesis and covalent inhibitors, *Digit. Discov.*, 2024, **3**, 347–354), we introduce an atom-based machine learning (ML) approach for predicting methyl cation affinities (MCAs) and methyl anion affinities (MAAs) to estimate nucleophilicity and electrophilicity, respectively. The ML models are trained and validated on QM-derived data from around 50 000 neutral drug-like molecules, achieving Pearson correlation coefficients of 0.97 for MCA and 0.95 for MAA on the held-out test sets. In addition, we demonstrate the ML approach on two different applications: first, as a general tool for filtering retrosynthetic routes based on chemical selectivity predictions, and second, as a tool for assessing the chemical stability of esters and carbamates towards hydrolysis reactions. The code is freely available on GitHub under the MIT open source license and as a web application at https://www.esnuel.org.

## Abstract 2

Nucleophilicity and electrophilicity are important properties for evaluating the reactivity and selectivity of chemical reactions.

## Graphical abstract

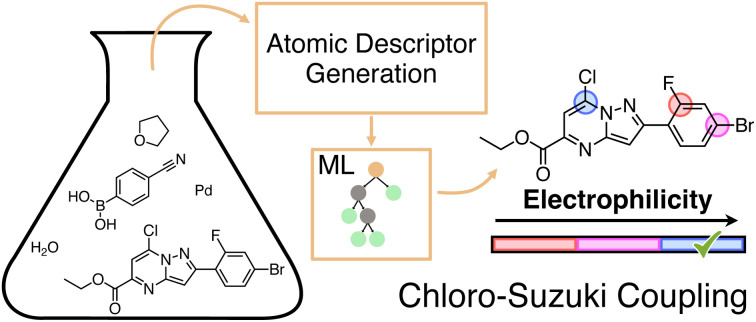

## Introduction

The interaction between electrophiles (electron-accepting species) and nucleophiles (electron-donating species) is a fundamental concept in chemistry for describing the reaction of molecules. The concept introduced by Ingold in 1933 is based on earlier theories of valency and acid–base chemistry,^1–4^ and it provides an important language for explaining chemical reactivity and selectivity. For example, Mayr and co-workers have shown that the reactivity of nucleophiles and electrophiles can be quantified on scales that describe their relative reactivity.^5^ Specifically, the Mayr–Patz equation links the bimolecular rate constant of various organic reactions to experimentally derived parameters such as nucleophilicity (*N*), electrophilicity (*E*), and a nucleophile-specific sensitivity factor (*s*_*N*_):

1 log *k*_20°C_ = *s*_*N*_(*N* + *E*)

Essential to eqn (1) is that it covers a broad range of reaction rates, from those that are virtually undetectable (*k*_20°C_ < 10^−5^ M^−1^ s^−1^) to those that are diffusion-controlled (*k*_20°C_ > 10^9^ M^−1^ s^−1^).^6,7^ However, experimentally measuring these reactivity parameters can be quite labor-intensive as well as difficult for reactions at the extreme ends of the reactivity scale. To streamline this process, various computational approaches have been developed. This includes estimating the rate constant using the Eyring equation^8,9^ and computing the reactivity parameters from frontier molecular orbital (FMO) energies^10,11^ or chemical affinities.^12–14^ Moreover, several machine learning (ML) approaches have recently emerged based on Mayr's database which currently holds experimental reactivity parameters for 355 electrophiles and 1300 nucleophiles.^6,15–21^

Our recent work introduces ESNUEL,^22^ a fully automated quantum chemistry (QM)-based workflow for EStimating NUcleophilicity and ELectrophilicity. A workflow that builds upon studies by van Vranken and Baldi showing that calculated methyl cation affinities (MCAs) and methyl anion affinities (MAAs) of structurally different molecules correlate with Mayr's N × *s*_*N*_ and *E*, respectively, when accounting for solvent effects.^13,14^ While ESNUEL provides good agreement^22^ with Mayr's experimental values (*R*^2^ = 0.84 and 0.94) and Baldi and van Vrankens computational results (*R*^2^ = 0.98 and 0.99), and provides excellent generalizability with a median wall time of less than two minutes per molecule using eight CPU cores for 2341 molecules (averaging ∼10 heavy atoms and ∼6 identified electrophilic and nucleophilic sites), further reducing the wall time to seconds or sub-seconds would be advantageous for many applications such as computer-aided synthesis planning (CASP).

In this work, we introduce two new atom-based ML models designed to predict MCA and MAA values for estimating nucleophilicity and electrophilicity. Our ML models are trained on QM-calculated MCA and MAA values of neutral drug-like molecules to ensure that the predictions apply to pharmaceutical research. Furthermore, the ML predictions are accompanied by reliable uncertainty estimates, which provide valuable information on when to employ the QM-based workflow. Compared to our QM-based workflow, the ML models significantly reduce the computational cost, achieving a median wall time of 0.36 seconds per molecule for the same 2341 molecules on a single CPU core. This makes the ML approach particularly useful as a post-filtering method in retrosynthesis planning, where it can quickly detect potential selectivity issues and thereby improve synthetic route design.

## Methods

### Dataset preparation

We employ a subset of 50 000 unique molecules from the ChEMBL database constructed to cover a large part of the drug-relevant chemical space. The subset is limited to neutral closed shell molecules with a maximum number of 24 rotatable bonds (median = 6.0, mean = 6.3) and 10 to 30 heavy atoms (median = 24.0, mean = 23.7). Using our recently introduced QM-based workflow,^22^ we detect nucleophilic and electrophilic atomic sites for each molecule and compute their corresponding methyl cation affinities (MCAs) and methyl anion affinities (MAAs) according to eqn (2) and (3). The only change to the original QM-based workflow is that for all reactants and products, we embed 20 conformers using RDKit^23^ instead of min(1 + 3 × *n*_rot_, 20) conformers, where *n*_rot_ is the number of rotatable bonds. The MCAs and MAAs are obtained at the r^2^SCAN-3c SMD(DMSO)//GFN1-*x*TB ALPB(DMSO) level of theory. An overview of the QM-based workflow is presented in Fig. S1 in the ESI.†2
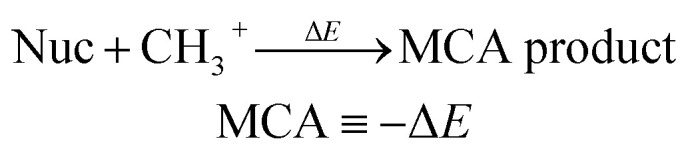
3
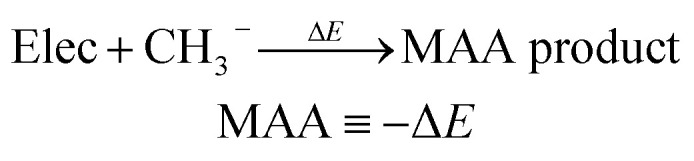


After completing the QM calculations, the results are categorized into two datasets: one for nucleophilic sites with corresponding MCA values, and the other for electrophilic sites with their MAA values. Calculations that lead to changes in the atom connectivity are excluded. Such connectivity changes typically arise from unwanted proton transfers or fragmentation reactions during the geometry optimization. Additionally, six MCA and one MAA calculations are excluded based on Chauvenet's criterion, where the probability of the most extreme MCA or MAA value is calculated under the assumption of a Gaussian distribution. If the probability falls below a predefined threshold of 1%, the point is removed, and the process is repeated until all points are above the threshold. Consequently, the MCA dataset consists of 650 857 unique atomic sites from 47 921 unique molecules, while the MAA dataset includes 534 119 unique atomic sites from 47 440 unique molecules. Distributions of MCA and MAA values, as well as the numbers for various detected functional groups, can be found in the ESI.†

### Atomic descriptors

**Fig. 1:**
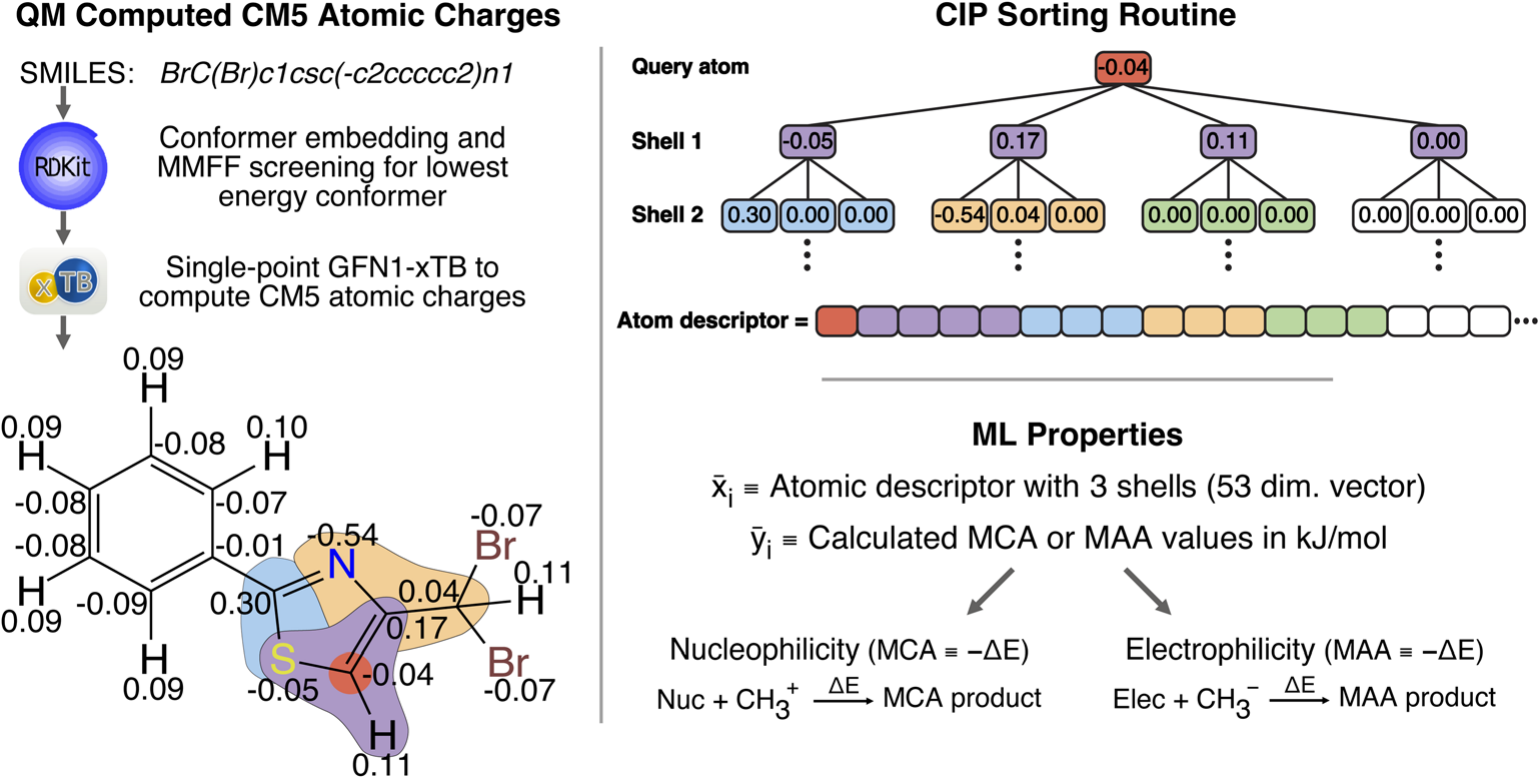
The workflow for creating atomic descriptors used as input feature vectors for machine learning. The atomic descriptors consist of charge model 5 (CM5) atomic charges sorted according to a modified version of the Cahn–Ingold–Prelog (CIP) rules. The target values are either MCA or MAA values at the r^2^SCAN-3c SMD(DMSO)//GFN1-*x*TB ALPB(DMSO) level of theory.

Following previous work on atomic property predictions,^24–31^ we construct a 53-dimensional feature vector of sorted atomic charges for each identified nucleophilic and electrophilic atomic site as seen in Fig. 1.

Starting from a SMILES string of a given molecule, we embed min(1 + 3 × *n*_rot_, 20) conformers using RDKit, where *n*_rot_ is the number of rotatable bonds. Each conformer then undergoes a geometry optimization using the Merck molecular force field (MMFF, version MMFF94s) implemented in RDKit.^32,33^ This allows us to extract the lowest energy conformer for which we calculate charge model 5 (CM5) atomic charges by running a single point calculation using GFN1-*x*TB as implemented in the open source semiempirical software package *x*TB.^34,35^ While the CM5 charge scheme has been shown to be largely conformation-independent^24^ we found that the MMFF optimisation has a small positive effect on the accuracy of the ML model, and decided to include this step in the workflow as it has negligible computational cost. The nucleophilic and electrophilic atomic sites are then found by matching a set of SMARTS patterns using RDKit. All of the SMARTS patterns for the nucleophilic and electrophilic atomic sites are provided in the associated GitHub repository.

The nucleophilic sites include double/triple-bonded atoms, singly charged anions, atoms with lone pairs, and specific functional groups such as aldehydes, amides, amines, carbanions, carboxylic acids, cyanoalkyl/nitrile anions, enolates, esters, ethers, imines, isonitriles, ketones, nitranions, nitriles, and nitronates.

The electrophilic sites include double/triple-bonded atoms, singly charged cations, and specific functional groups such as acyl halides, aldehydes, amides, anhydrides, boranes, carbocations, esters, imines, iminium ions, ketones, Michael acceptors, and oxonium ions.

Finally, the atomic descriptors are constructed for the identified atomic sites by creating and concatenating shells of neighboring atoms around the query atom, including atoms up to three bonds away. The shells contain calculated CM5 atomic charges sorted according to a modified version of the Cahn–Ingold–Prelog (CIP) priority rules; modifications include summing atomic numbers rather than comparing sorted lists of atomic numbers, and disregarding bond orders. Compared to our previous work, we have re-implemented the sorting algorithm to comply with the following priority rules:

1 Sort according to atomic number in descending order.

2 If (1) is not unique, for each atom with the same priority (A*):

(i) Go to bound and yet not included atoms and sum up atomic numbers. Set the priority of A* according to the sum of the atomic numbers.

(ii) If (2i) did not give an unambiguous result expand the shell of each atom A* by one bond.

(iii) Repeat (2ii) until a unique order is found.

3 If no unique order is found in (2) and all bound atoms are included, then sort atoms according to the CM5 charges in descending order.

### Machine learning model

After introducing the target values and feature vectors, we now divide the MCA and MAA datasets into training and held-out test sets using a binned split approach, with 85% of the data allocated for training and 15% reserved for testing. Specifically, this results in a training and held-out test set of 553 228 and 97 629 atomic sites for the MCA model and 454 001 and 80 118 atomic sites for the MAA model. The binned split ensures that the distribution of target values as well as the partitioning of functional groups is consistent across both sets as seen in the ESI.†

For predicting MCA and MAA values, we train a light gradient boosting machine (LightGBM) regression model for each property.^36^ The LightGBM model is chosen based on previous benchmark studies of several ML models in combination with the atom-based feature vectors.^30^ The hyperparameters for LightGBM models are obtained using a tree-structured Parzen estimator (TPE) as implemented in Optuna version 2.5.0.^37^ This Bayesian optimization method efficiently explores a large hyperparameter space to identify the configurations that minimize the root mean square error (RMSE). For each set of hyperparameters as well as the final model training, we conduct a 5-fold cross-validation to ensure a robust model performance. The cross-validation involves a stratified split of the binned training set, which helps maintain the distribution of target values across the folds, and we apply random shuffling to avoid any bias in the data. This ensures that the model is evaluated on diverse subsets of the data, providing a more comprehensive assessment of its predictive performance. After the final model training, we only retain the model with the lowest RMSE from the 5-fold cross-validation.

In addition, we train a random forest regression model with 200 estimators as implemented in scikit-learn.^38^ By accessing the trained estimators in the random forest model, we can obtain a target prediction for all 200 decision trees and calculate a standard deviation. This standard deviation is then used to estimate the prediction uncertainties of the final LightGBM model and to forecast out-of-sample data points where running the QM-based workflow would be recommended. The performance of this uncertainty estimation approach is evaluated using error-based calibration following the work of Rasmussen *et al.*^39^ Error-based calibration is an uncertainty quantification (UQ) metric proposed by Levi *et al.*^40^ based on the principle that the root mean square error (RMSE) should directly correlate with the root mean variance (RMV). To explore the local relationship between the predicted uncertainties (*σ*) and errors (*ε* = *y*_pred_ − *y*_true_) of the ML predictions (*y*_pred_) compared to the QM calculations (*y*_true_), the two properties are sorted and binned according to the uncertainty. For each bin containing *N*_bin_ samples, RMSE and RMV are calculated as follows:4
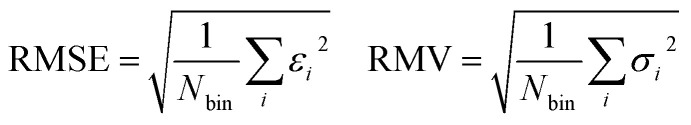


A plot of RMSE against RMV should ideally yield a straight line with a slope of one and an intercept of zero.

Additional ML models for predicting the MCA and MAA values and estimating uncertainties have also been explored including ensemble models and *k*-nearest neighbor models with *k* = {1,10}. The performance of these methods is provided in the ESI.†

## Results and discussion

In this section, we begin by assessing the performance of the two ML models and the ability to provide a confidence value for ML predictions. Following this, we will explore potential applications such as using chemical selectivity predictions to post-filter retrosynthetic routes and evaluating the chemical stability of esters and carbamates towards hydrolysis reactions.

### Machine learning model performance

**Fig. 2:**
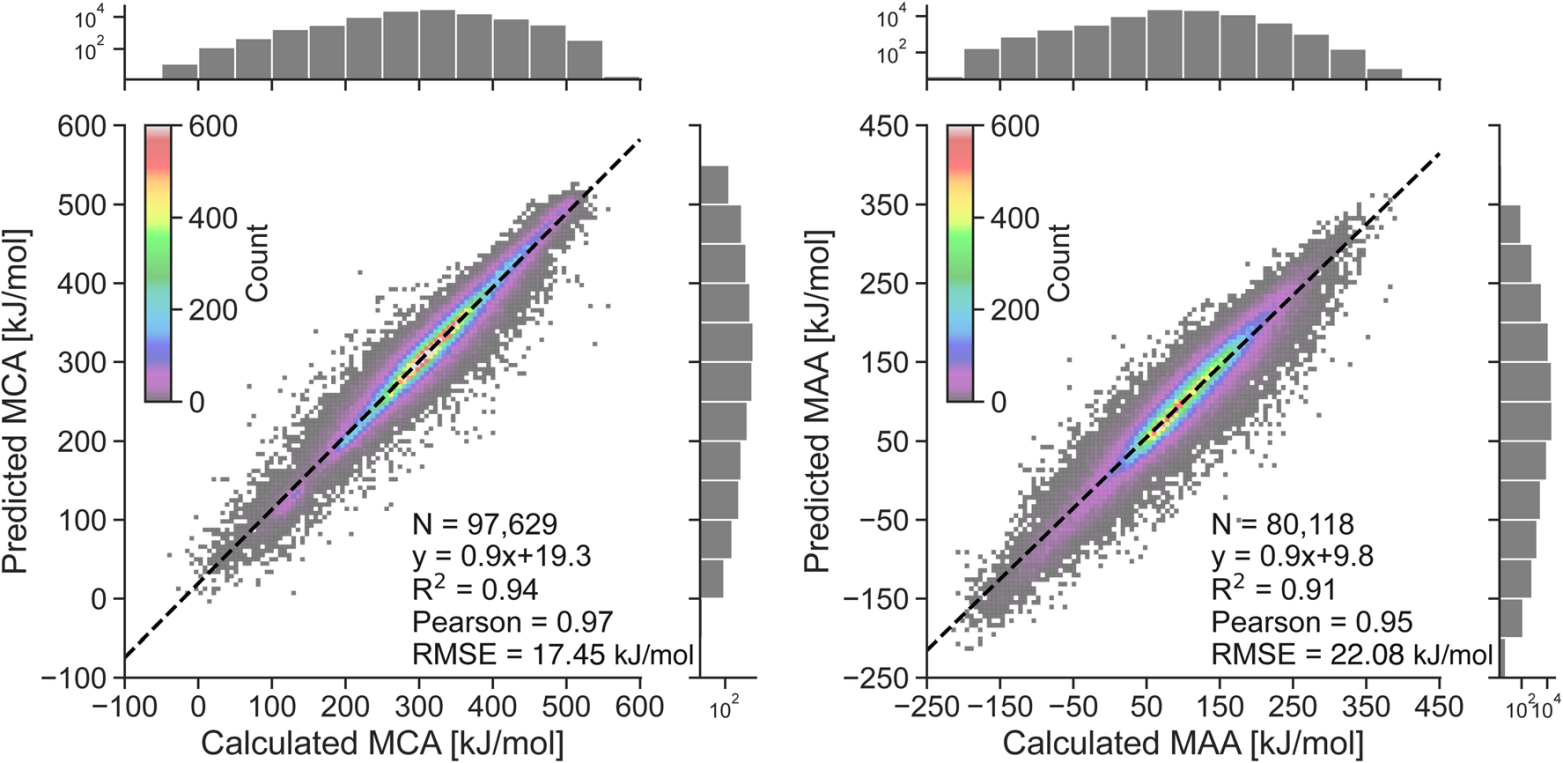
Correlation plots comparing the QM-calculated and ML-predicted MCA and MAA values for held-out test sets. The color grading represents the density of data points, while the black dashed line represents the linear regression. The calculated MCA and MAA values are obtained at the r^2^SCAN-3c SMD(DMSO)//GFN1-*x*TB ALPB(DMSO) level of theory.

The performance of the two atom-based ML models for estimating nucleophilicity and electrophilicity, through the prediction of MCA and MAA values, is shown in Fig. 2. The results show a strong correlation between QM-calculated and ML-predicted MCA and MAA values with Person correlation coefficients of 0.97 and 0.95, respectively. Note, that the color grading represents a 2D histogram of MCA and MAA values, showing that these values are mainly centered around the black regression line. The RMSE on the held-out test sets is 17.45 kJ mol^−1^ for the MCA model and 22.08 kJ mol^−1^ for the MAA model, with corresponding MAE values of 11.93 kJ mol^−1^ and 15.32 kJ mol^−1^. These values are consistent with the 5-fold cross-validation results with RMSE of 17.56 ± 0.05 kJ mol^−1^ and 22.17 ± 0.03 kJ mol^−1^ for the MCA and MAA model, respectively.

**Fig. 3:**
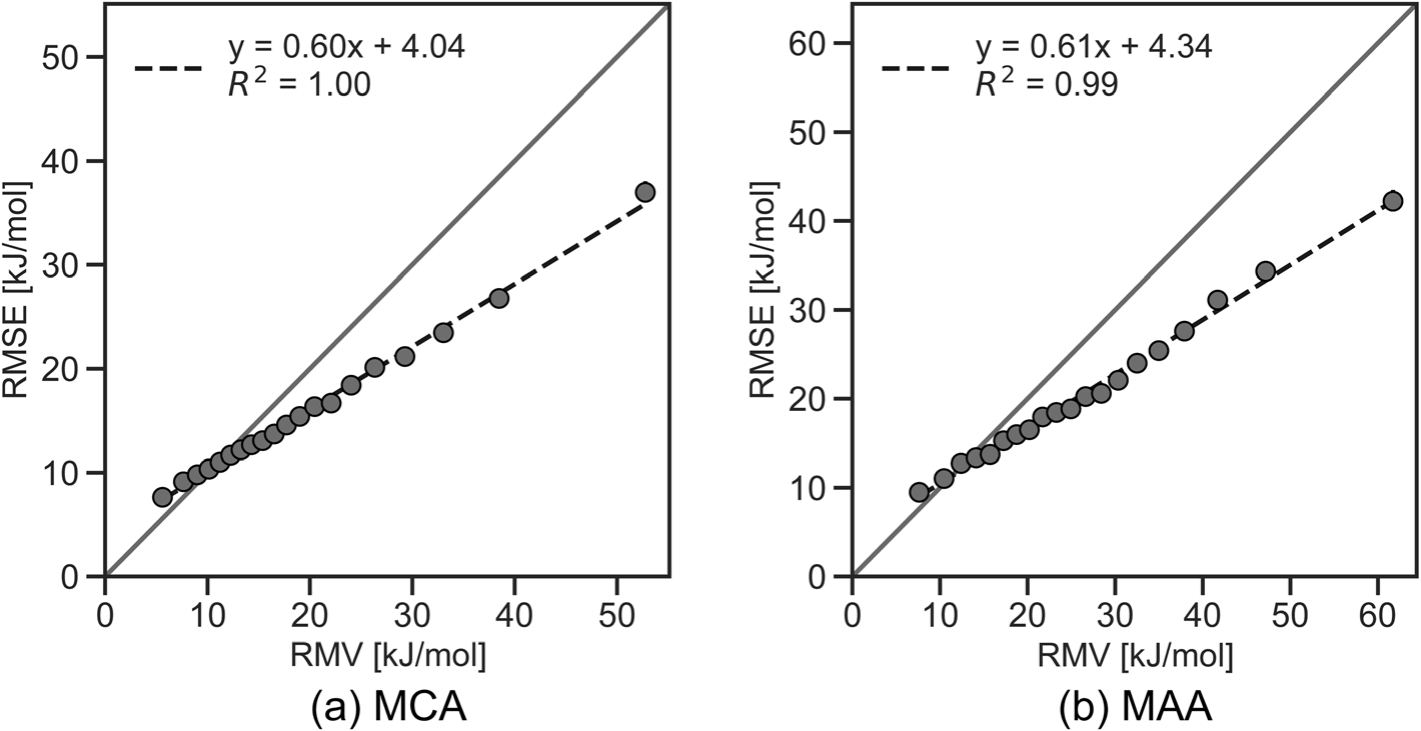
Error-based calibration plots of (a) MCA and (b) MAA held-out test sets. The uncertainty estimates are based on the standard deviation of 200 decision trees from the random forest regression models trained on the MCA and MAA training sets. For well-calibrated uncertainties, the root mean square error (RMSE) *vs.* root mean variance (RMV) plot should follow a straight line with a slope of one and an intercept of zero.

As seen in Fig. 2, a few data points result in relatively large errors exceeding 100 kJ mol^−1^, specifically 136 for MCA dataset and 241 for MAA dataset. To detect such outliers and estimate the uncertainties of the LightGBM model predictions, we have explored different UQ methods such as the random forest standard deviation, the ensemble model standard deviation, and the average feature vector distance (Manhattan or Euclidean) to the *k* = {1,10} nearest data points in the training set as seen in the ESI.† These UQ methods are validated using the error-based calibration metric by Levi *et al.*^40^ following the work of Rasmussen *et al.*^39^ The results clearly show that the most reliable uncertainty estimates are obtained using the random forest standard deviation as seen in the ESI.† The error-based calibration plots for the MCA and MAA held-out test sets using the random forest standard deviation are shown in Fig. 3 highlighting a direct relationship between the RMSE and RMV with *R*^2^ > 0.99.

**Fig. 4:**
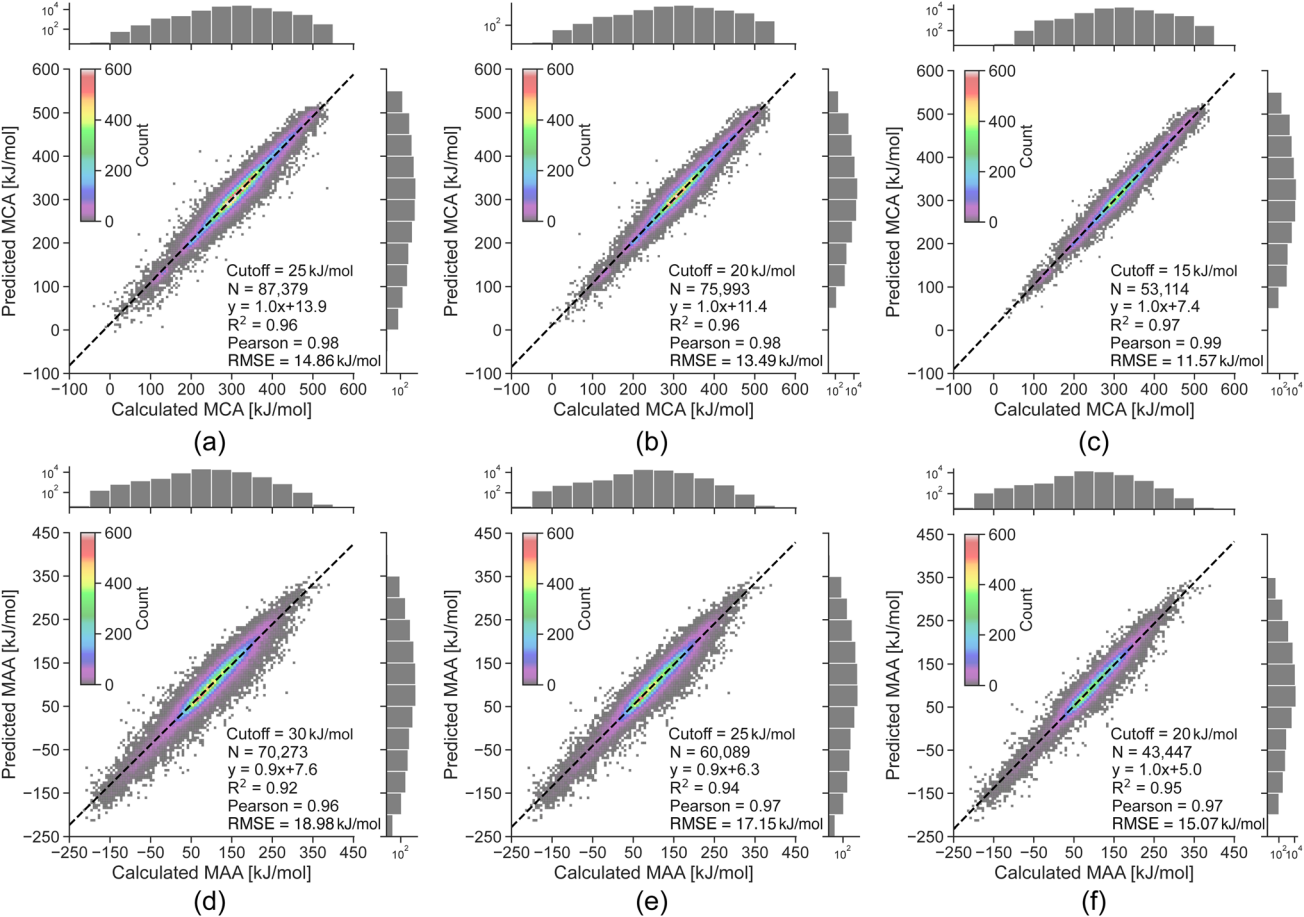
Correlation plots comparing the QM-calculated and ML-predicted MCA and MAA values for held-out test sets with the effect of removing predictions having an estimated error above the specified cutoff. The color grading represents the density of data points, while the black dashed line represents the linear regression. The calculated MCA and MAA values are obtained at the r^2^SCAN-3c SMD(DMSO)//GFN1-*x*TB ALPB(DMSO) level of theory.

The linear regression equations in Fig. 3 can be used to convert the predicted uncertainties from the random forest model into an estimated error of the LightGBM model predictions. However, as the data is binned according to the uncertainty, the results only reflect the general trend of a higher predicted uncertainty leading to a higher probability of a large predicted error. Thus, a high predicted uncertainty can still result in a low error. The main objective of the uncertainty predictions is therefore not to accurately estimate errors, but rather to serve as a tool for identifying potentially incorrect property predictions, where it would be advisable to run the QM-based workflow. To achieve this, we define estimated error cutoffs based on the amount of QM calculations we allow to compute for the held-out test set. For estimated errors above 25 kJ mol^−1^ for MCA and 30 kJ mol^−1^ for MAA, we permit around 10% of the held-out test set with the highest predicted uncertainties to be processed using the QM-based workflow. Returning to the data points with true errors exceeding 100 kJ mol^−1^, we can identify 110 out of 136 outliers for MCA (81%) and 164 out of 241 for MAA (68%) using these estimated error cutoffs. Reducing the cutoffs to 20 and 25 kJ mol^−1^ (∼25% of the held-out test set) will increase the detection rate to 93% for MCA and 82% for MAA, while cutoffs of 15 and 20 kJ mol^−1^ (∼45% of the held-out test set) allow the detection of 100% for MCA and 92% for MAA as seen in Fig. 4.

**Fig. 5:**
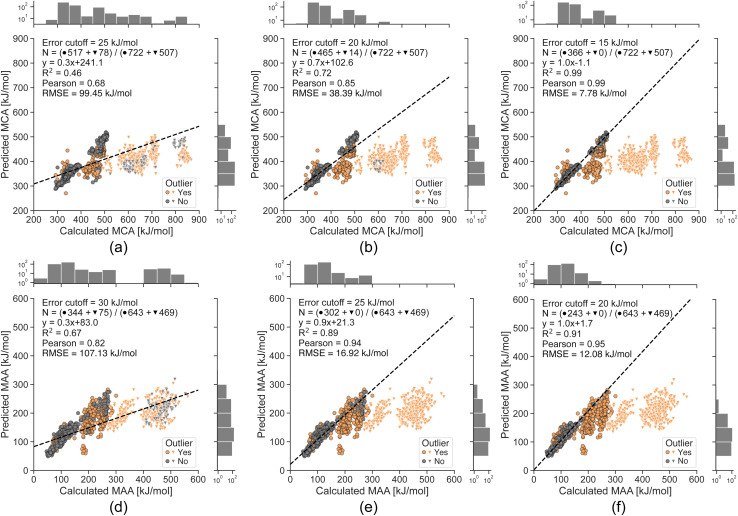
Correlation plots comparing the QM-calculated and ML-predicted MCA and MAA values for a non-drug-like dataset from Tavakoli *et al.*^41^ with the effect of removing predictions having an estimated error above the specified cutoff. The ML models are only trained on neutral drug-like molecules, and the bright triangles indicate compounds with a non-zero formal charge. The number of included data points in grey (*N*) is specified in each plot along with the total number of neutral compounds (●) and compounds with a non-zero formal charge (▼). The calculated MCA and MAA values are obtained at the r^2^SCAN-3c SMD(DMSO)//GFN1-*x*TB ALPB(DMSO) level of theory.

To explore the limits of our ML models, we now evaluate them on a challenging non-drug-like dataset from Tavakoli *et al.*^41^ containing both neutral compounds and compounds with a non-zero formal charge (see Fig. 5). The ML models suffer from large errors for the small molecules with a non-zero formal charge (*i.e.* protonated or deprotonated molecules), which can be explained by the fact that the ML training sets only contain neutral drug-like molecules. However, we can detect most of these data points using the error estimates with cutoffs of 25 and 30 kJ mol^−1^ for MCA and MAA, respectively, and all of them with strict cutoffs of 15 and 20 kJ mol^−1^ for MCA and MAA, respectively. The Pearson correlation between the QM-calculated and ML-predicted MCA and MAA values goes from 0.68 to 0.99 for MCA and 0.82 to 0.95 for MAA. This further validates the ability to reliably identify outliers and shows that lower estimated errors reflect greater confidence in the predicted properties.

### Selectivity predictions for computer-assisted retrosynthesis

**Fig. 6:**
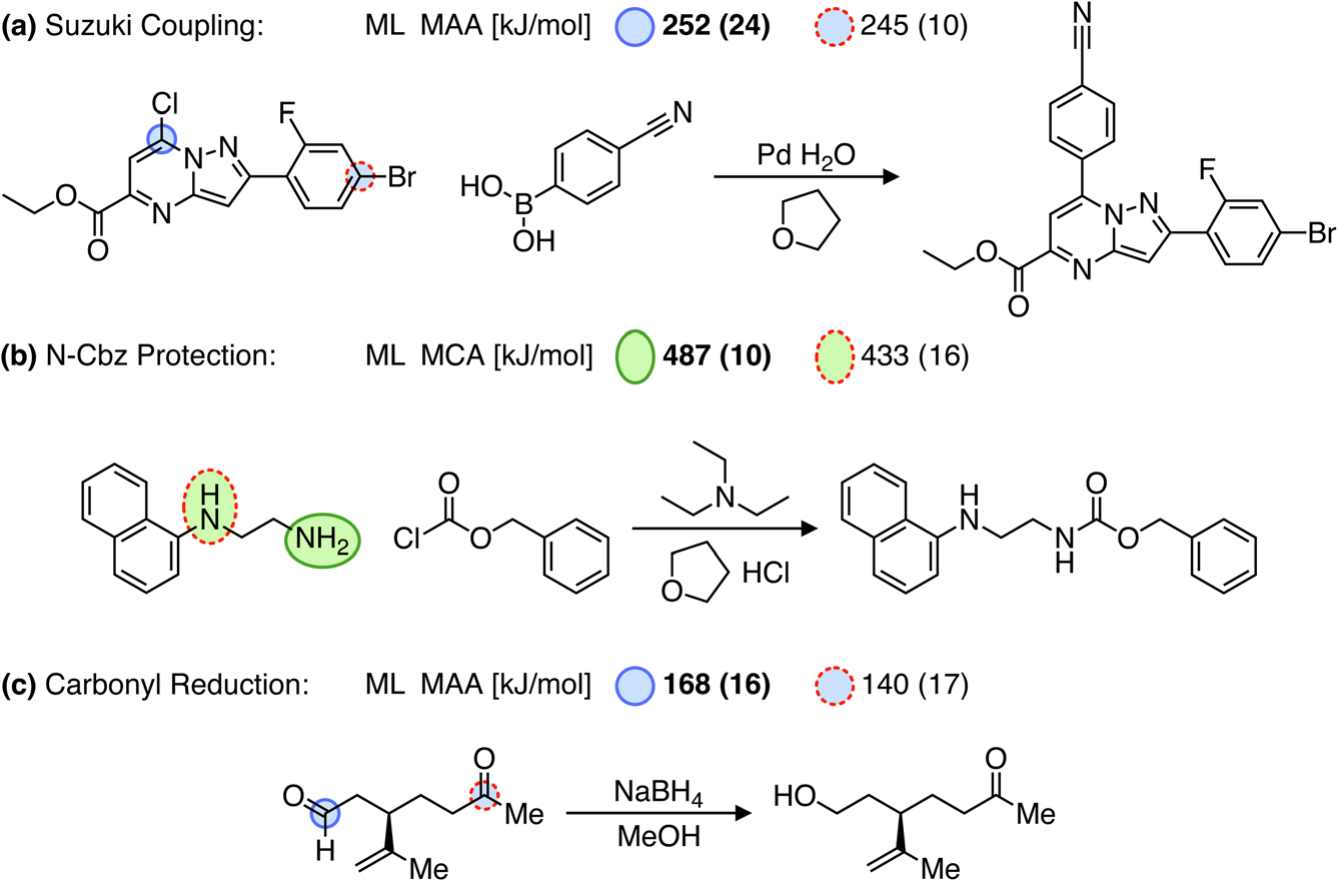
Reactions (a and b) from Joung *et al.*^42^ and (c) from Strieth-Kalthoff *et al.*,^43^ where predicting the selectivity can be a challenge for data-driven reaction prediction models. Note that only the recorded products are shown. The values in the parentheses are the estimated errors used to detect outliers, where running the QM-based workflow would be recommended. However, all values are below the predefined cutoffs of 25 and 30 kJ mol^−1^ for MCA and MAA, respectively.

Over the past few years, significant progress has been made in computer-aided synthesis planning (CASP) leading to highly promising tools for accelerating the synthesis of chemical compounds and providing valuable feedback to generative models on the synthesizability of suggested molecules. However, one of the major challenges for CASP tools is their ability to account for chemical selectivity.^42,43^ A recent paper even emphasizes the need for incorporating expert knowledge (*i.e.* reaction rules) into data-driven CASP tools to address the selectivity problems. Although different reaction conditions can alter the chemical selectivity and such “condition matching would require an extensive study by expert chemists and would likely only be applicable for extremely simple reaction types”.^43^ As an alternative to reaction rules, we suggest using MCA and MAA values to assess the relative reactivity between reaction sites. This approach is demonstrated in Fig. 6 for reaction examples where predicting the selectivity poses a challenge for data-driven methods.^42,43^

For example, in a Suzuki coupling reaction involving two competing aryl halides as seen in Fig. 6a, a Graph2SMILES model from the Coley group can recover pathways for both bromo- and chloro-coupling products. However, the model incorrectly ranks the bromo product as more likely, even though the chloro product is the recorded outcome.^42^ As this reaction relies on the electrophilicity of the carbon atom adjacent to the halide for reacting with a palladium catalyst, we use our MAA ML model to find the most electrophilic site. As seen in Fig. 6a, the MAA ML model successfully predicts the recorded chloro-coupling product given the higher MAA value of 252 kJ mol^−1^ for the chloro site compared to 245 kJ mol^−1^ for the bromo site.

In another example, we explore an N-Cbz protection reaction using benzyl chloroformate as seen in Fig. 6b. Here, Joung *et al.*^42^ have shown that both a Weisfeiler–Lehman difference network (WLDN) and a transformer model can correctly predict the recorded product. In our approach, we will use the MCA ML model to identify the most nucleophilic site in *N*-(1-naphthyl)ethylenediamine. Using the MCA values, we can successfully predict the primary amine as the most reactive site with an MCA value of 487 kJ mol^−1^ compared to 433 kJ mol^−1^ for the secondary amine.

Finally, we explore a carbonyl reduction reaction with NaBH_4_, where the distinction between aldehyde and ketone is familiar to chemists but can be challenging to infer from literature examples.^43^ The reaction proceeds *via* a nucleophilic addition of borohydride to the carbonyl carbon, which therefore depends on the electrophilicity of the carbonyl group. Hence, we use the MAA ML model to predict the most electrophilic site of the reactant. As seen in Fig. 6c, we can successfully predict the aldehyde as the most reactive site given the higher MAA value of 168 kJ mol^−1^ compared to 140 kJ mol^−1^ for the ketone.

**Fig. 7:**
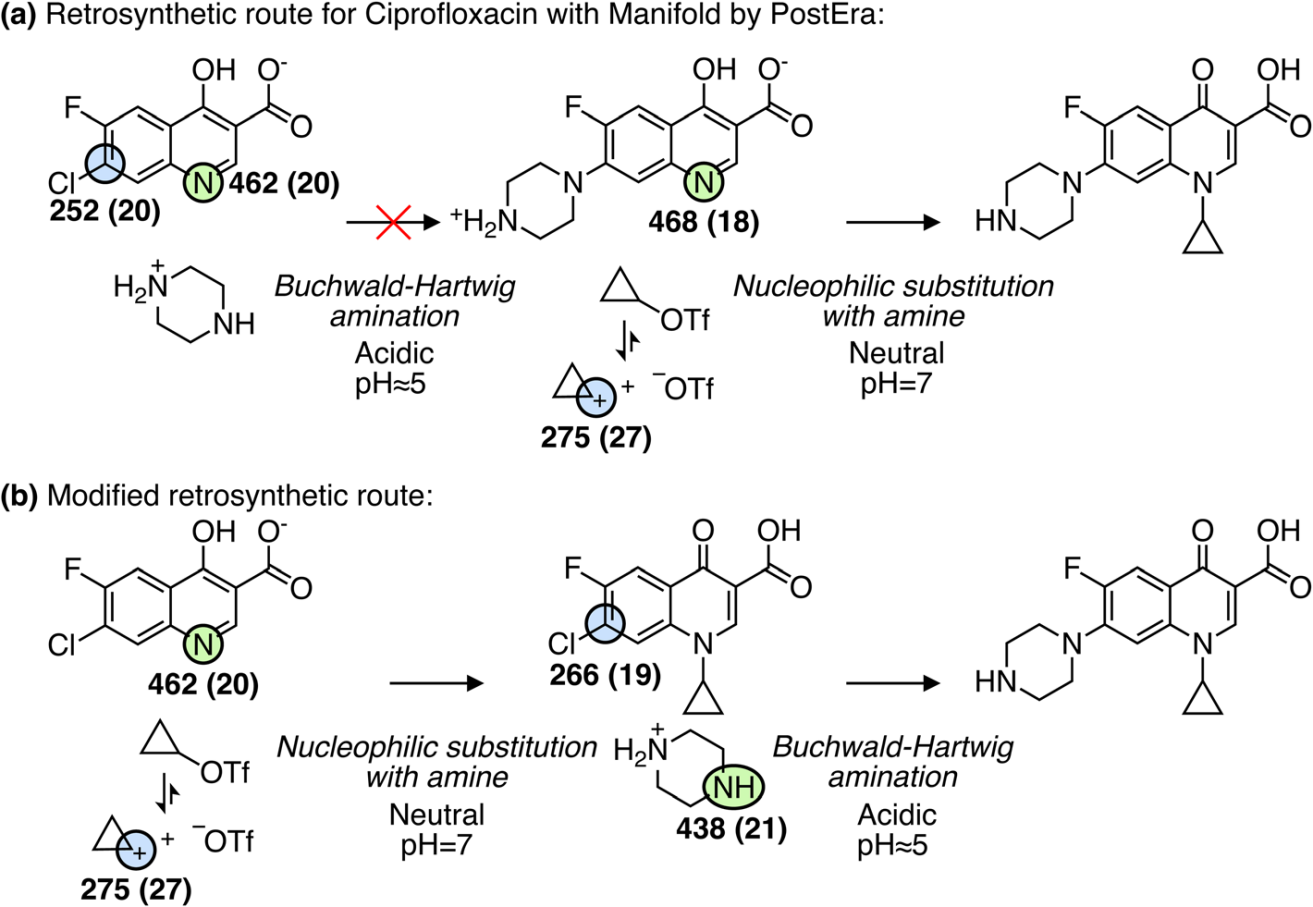
(a) Retrosynthesis predictions for ciprofloxacin using manifold by PostEra with the ML-predicted MCA values (green) and MAA values (blue) in units of kJ mol^−1^. Only the most nucleophilic and electrophilic sites are highlighted. The values in the parentheses are the estimated errors used to detect outliers, where running the QM-based workflow would be recommended. However, all values are below the predefined cutoffs of 25 and 30 kJ mol^−1^ for MCA and MAA, respectively. (b) Same as for (a) but for a modified retrosynthetic route.

Having demonstrated that our ML models can successfully predict chemical selectivity in challenging examples, we now explore how the ML models can improve retrosynthetic route predictions by identifying potential selectivity issues. Fig. 7a shows a synthetic pathway for ciprofloxacin generated using Manifold by PostEra.^44^ All structures are presented in their major protonation state as determined by MarvinSketch based on the expected reaction conditions. The predicted p*K*_a_ values and protonation states at different pH levels are shown in the ESI.† For the first reaction step in Fig. 7a, the ML models predict the most nucleophilic and electrophilic sites to be in the quinoline derivative. This could lead to an undesired product where the quinoline derivative reacts with itself instead of reacting with piperazin-1-ium as proposed by Manifold. The second most nucleophilic site is the neutral nitrogen atom in piperazin-1-ium with an MCA value of 438 kJ mol^−1^ compared to 462 kJ mol^−1^ for the most nucleophilic site. As a result, this could lead to low yields of the target product, if this reaction occurs. The chemoselectivity of the second reaction step is correct according to the ML predictions. The assumption here is that triflate acts as a good leaving group forming a cyclopropyl cation, which will have the highest MAA value. The most nucleophilic site is the nitrogen atom in the quinoline derivative highlighted in green in Fig. 7a. However, as this route has potential selectivity issues, we suggest a modified retrosynthetic route as shown in Fig. 7b. Here, the two reaction steps are switched around such that the nucleophilic substitution with amine is the first reaction step and the Buchwald–Hartwig amination is the second reaction step. In this modified retrosynthetic route, the ML-predicted MAA and MCA values agree with the suggested chemoselectivity. In fact, the second reaction step in Fig. 7b combining fluoroquinolonic acid and piperazine is reported in the literature with a high yield of around 90% under acidic conditions.^45,46^

### Prediction of hydrolysis rates for determining chemical stability

**Fig. 8:**
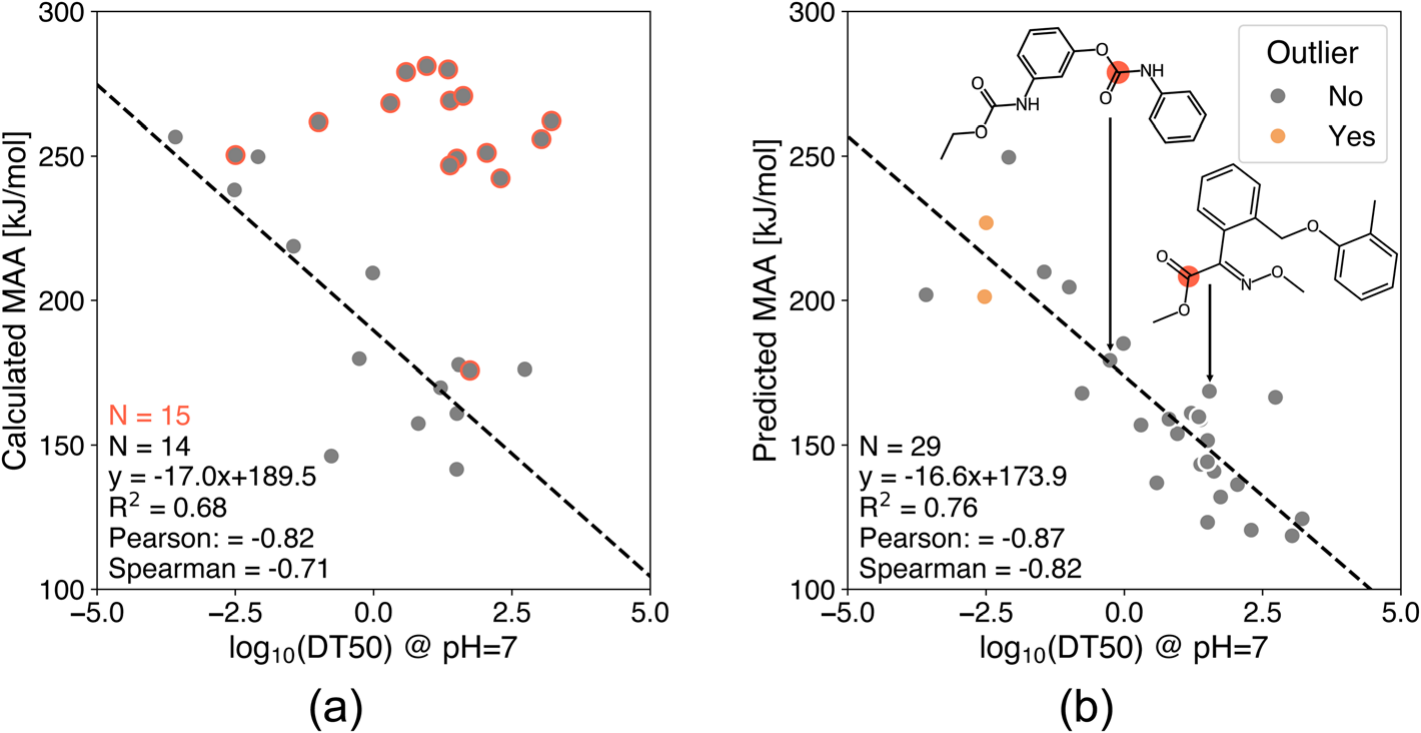
Correlation plots of (a) QM-calculated and (b) ML-predicted MAA values against experimental hydrolysis half-lives of 29 esters and carbamates under neutral pH.^47^ The red circles indicate a connectivity change during the geometry optimization and are therefore excluded from the analysis. The calculated MAA values are obtained at the r^2^SCAN-3c SMD(DMSO)//GFN1-*x*TB ALPB(DMSO) level of theory. The data points marked in orange have an estimated error above the predefined cutoff of 30 kJ mol^−1^.

**Fig. 9:**
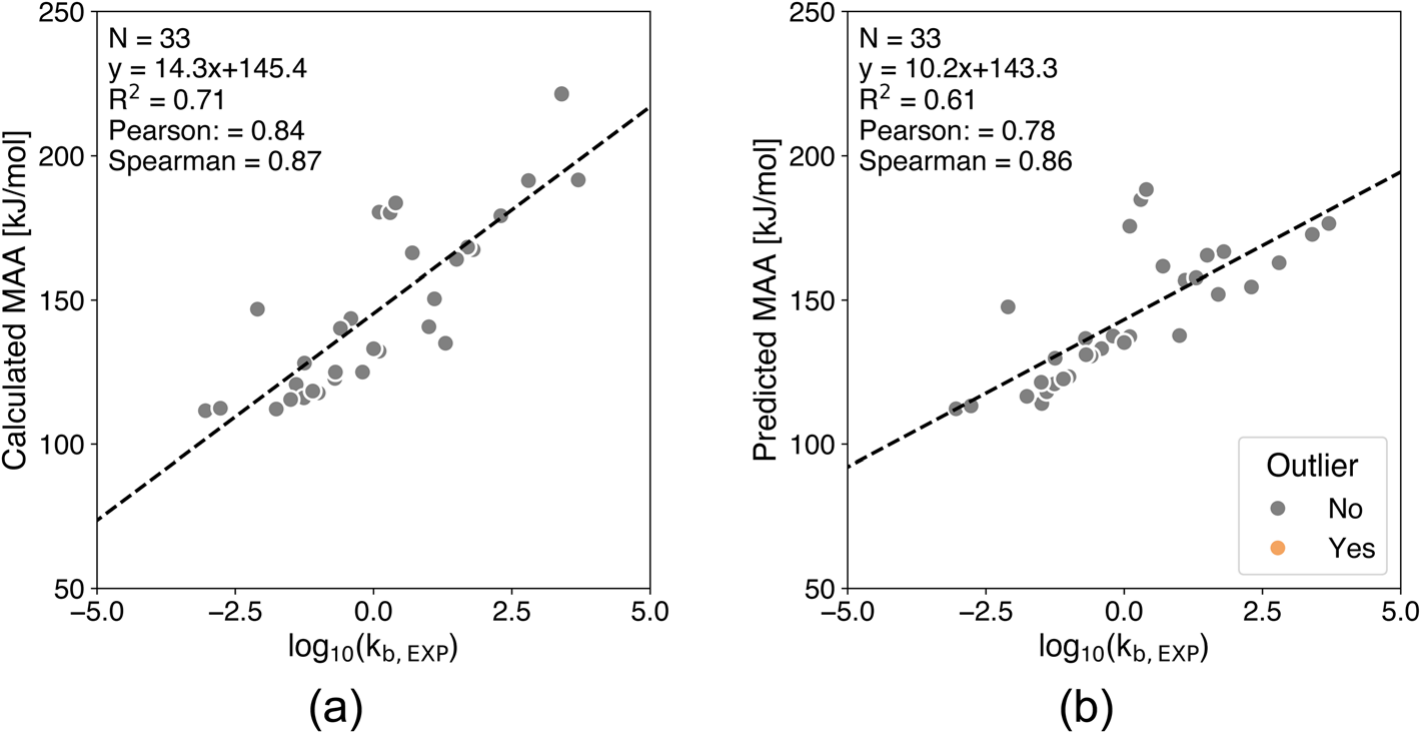
Correlation plots comparing (a) QM-calculated and (b) ML-predicted MAA values against experimental hydrolysis rates for 33 esters and carbamates under basic conditions.^48–50^ The calculated MAA values are obtained at the r^2^SCAN-3c SMD(DMSO)//GFN1-*x*TB ALPB(DMSO) level of theory. All of the predicted MAA values have estimated errors below the predefined cutoff of 30 kJ mol^−1^.

Another application of the presented ML models is to determine chemical stability by applying the MAA values to predict hydrolysis half-lives or rates of esters and carbamates. Fig. 8 and 9 show the correlation between MAA values and experimental hydrolysis half-lives and rates of esters and carbamates under neutral and basic conditions, respectively.^47–50^ The MAA values are obtained for the neutral species without adjusting the protonation state to the reaction conditions, and only the maximum MAA value is used if multiple ester and carbamate groups are present in the same molecule. The results in Fig. 8 show the correlation between QM-calculated and ML-predicted MAA values against experimental hydrolysis half-lives of 29 esters and carbamates under neutral pH. Unfortunately, 15 of the QM calculations resulted in connectivity changes during the geometry optimizations as indicated by the red circles in Fig. 8a. The reasons for the connectivity issues include hydrogen transfers, cyclization, and fragmentation of the starting structure. Consequently, the MAA values are generally too high for the MAA calculations with connectivity issues, and these results are therefore excluded from the analysis as seen in Fig. 8a. In contrast, the ML model does not encounter this problem as the molecular geometries are optimized using MMFF. The Pearson correlation coefficient for all 29 compounds using the ML model is −0.87 compared to the QM-calculated MAA values with a Pearson correlation coefficient of −0.82 for the 14 compounds without connectivity issues. As seen in Fig. 8b, two of the ML-predicted MAA values had an estimated error above the predefined 30 kJ mol^−1^ cutoff. However, both of these data points are in good agreement with the regression line.

For the base-promoted hydrolysis of 33 esters and carbamates shown in Fig. 9, the Pearson correlation coefficients between the experimental hydrolysis rates and the QM-calculated or ML-predicted MAA values are 0.84 and 0.78, respectively. This reflects a strong correlation for both methods, although the slope of the regression line for the QM calculations is slightly steeper than for the ML-predicted MAA values, leading to greater separation between data points for the QM-calculated MAA values. All of the ML-predicted MAA values had an estimated error below the predefined cutoff of 30 kJ mol^−1^, thus no outliers were identified.

The strong correlations in Fig. 8 and 9 show the potential of using the ML-predicted MAA values to predict the hydrolysis half-lives or rates of esters and carbamates despite the relatively small range in MAA values. Thus, supporting the use of the MAA ML model to guide the design of esters and carbamates by making them less prone to undesired hydrolysis reactions and thereby improving their chemical stability.

## Conclusions and outlook

We present two atom-based machine learning (ML) models for estimating nucleophilicity and electrophilicity by predicting methyl cation affinities (MCAs) and methyl anion affinities (MAAs). The ML models are trained on quantum chemistry (QM)-calculated data of neutral drug-like molecules generated with our recently introduced QM-based workflow.^22^ Thus, the ML models are designed to replicate QM-calculated MAA and MCA values, while significantly reducing the computational costs. The Pearson correlation coefficients between the QM-calculated and ML-predicted MCA and MAA values for the held-out test set are 0.97 and 0.95, respectively. In addition, the ML models are accompanied by uncertainty quantification (UQ) predictions, which enable the detection of out-of-sample data points. This UQ approach is intended to inform about when it is recommended to run the QM-based workflow to obtain reliable results. For example, we show that the approach can detect data points from small non-drug-like molecules with a non-zero formal charge, which resulted in large errors due to the molecules being quite different from the underlying training data.

The potential of the ML models is demonstrated through their accurate predictions of both chemical selectivity and reactivity. For example, we show that the ML models can successfully predict chemical selectivity in cases that are typically challenging for data-driven computer-aided synthesis planning (CASP) tools to learn. As a result, we propose to integrate these ML models into CASP tools to flag reaction steps that can lead to low yields or undesired products. The proposed synthetic routes can then be ranked based on the number of flags to ensure the selection of the most reliable synthetic pathways. It is important to note, however, that integrating these ML models into CASP tools would require additional information about the individual reaction steps. This includes information about the reaction condition and the reaction mechanism. Information that is usually available in the underlying data of the CASP tools, but typically not provided. For example, a factor like pH can affect the protonation state of a molecule, which can be crucial for the reaction to proceed and have a great impact on the nucleophilicity and electrophilicity estimates. To address this, the MCA and MAA models would have to be accompanied by a p*K*_a_ predictor to accurately determine the correct protonation state under the given reaction conditions. Alternatively, the CASP tools would need to provide the reactants in their actual protonation state instead of the neutral canonical form. Information about the reaction mechanism is another important factor for evaluating the chemical selectivity and determining the properties that drive the reaction. For example, if the reaction involves a catalyst, a single proposed reaction step could involve multiple key steps that influence selectivity such as the formation of a catalyst-substrate complex to unlock the true reaction site. Furthermore, knowledge about leaving groups is also important, as this can significantly impact the predicted MCA and MAA values. Unfortunately, the current CASP tools typically lack this level of reaction details and mechanistic insights. However, novel approaches for determining reaction mechanisms using ML have recently emerged,^42^ which could be a step in the right direction for making CASP tools truly selectivity-aware through the use of MCA and MAA values. In terms of reactivity predictions, we show that the ML-predicted MAA values can be used to estimate the hydrolysis half-lives or rates of esters and carbamates to guide the chemical design toward more stable compounds.

Future improvements include the use of different protonation states of the molecules in the datasets to expand the applicability domain of the ML models. This will affect the calculated atomic CM5 charges, and can therefore be easily incorporated by adding new data to the existing datasets. Additionally, the UQ approach could be leveraged to guide the inclusion of new data based on the estimated errors through an active learning procedure.

## Data availability

The data and code for developing and deploying the ML models as well as obtaining the presented results are available at https://github.com/jensengroup/ESNUEL_ML.

### Author contributions

All authors contributed to the conceptualization and method development. N. R. wrote all the code and performed all the calculations. All authors contributed to the data analysis. All authors read and approved the final manuscript.

## Conflicts of interest

The authors declare that there are no competing interests.
